# Diet-Related Alterations of Gut Bile Salt Hydrolases Determined Using a Metagenomic Analysis of the Human Microbiome

**DOI:** 10.3390/ijms22073652

**Published:** 2021-04-01

**Authors:** Baolei Jia, Dongbin Park, Byung Hee Chun, Yoonsoo Hahn, Che Ok Jeon

**Affiliations:** 1 State Key Laboratory of Biobased Material and Green Papermaking, School of Bioengineering, Qilu University of Technology (Shandong Academy of Sciences), Jinan 250353, China; baoleijia@cau.ac.kr; 2 Department of Life Science, Chung-Ang University, Seoul 06974, Korea; neuro1009@gmail.com (D.P.); cbh0813@naver.com (B.H.C.); hahny@cau.ac.kr (Y.H.)

**Keywords:** gut microbiome, secondary bile acids, dietary pattern, metagenomic cohorts, human health

## Abstract

The metabolism of bile acid by the gut microbiota is associated with host health. Bile salt hydrolases (BSHs) play a crucial role in controlling microbial bile acid metabolism. Herein, we conducted a comparative study to investigate the alterations in the abundance of BSHs using data from three human studies involving dietary interventions, which included a ketogenetic diet (KD) versus baseline diet (BD), overfeeding diet (OFD) versus underfeeding diet, and low-carbohydrate diet (LCD) versus BD. The KD increased BSH abundance compared to the BD, while the OFD and LCD did not change the total abundance of BSHs in the human gut. BSHs can be classified into seven clusters; Clusters 1 to 4 are relatively abundant in the gut. In the KD cohort, the levels of BSHs from Clusters 1, 3, and 4 increased significantly, whereas there was no notable change in the levels of BSHs from the clusters in the OFD and LCD cohorts. Taxonomic studies showed that members of the phyla Bacteroidetes, Firmicutes, and Actinobacteria predominantly produced BSHs. The KD altered the community structure of BSH-active bacteria, causing an increase in the abundance of Bacteroidetes and decrease in Actinobacteria. In contrast, the abundance of BSH-active Bacteroidetes decreased in the OFD cohort, and no significant change was observed in the LCD cohort. These results highlight that dietary patterns are associated with the abundance of BSHs and community structure of BSH-active bacteria and demonstrate the possibility of manipulating the composition of BSHs in the gut through dietary interventions to impact human health.

## 1. Introduction

Bile acids (BAs), which mainly include cholic acid (CA) and chenodeoxycholic acid (CDCA) in humans, are derived from cholesterol and are further conjugated with amino acids in hepatocytes. Conjugated BAs with both hydrophobic (lipid soluble) and polar (hydrophilic) faces are actively secreted into the small intestine to emulsify, solubilize, and transport lipids, such as fatty acids, cholesterol monoglycerides, and fat-soluble vitamins. The metabolism of BAs is associated with obesity, diabetes, gallbladder diseases, gastrointestinal diseases, liver diseases, and cardiovascular diseases [1]. The concentration of BAs is typically evaluated in patients with obesity or type 2 diabetes (T2D) [2]. In patients with non-alcoholic fatty liver disease (NAFLD) total fecal BA concentrations are generally elevated [3]. As metabolic regulators, BAs can bind to the farnesoid X receptor or Takeda G protein-coupled receptor 5 (TGR5) to regulate glucose metabolism, insulin sensitivity, and hepatic metabolism [4]. Alteration of the BA pool may contribute to the dysregulation of metabolic homeostasis in obesity, T2D, and NAFLD [5]. In addition, the levels of BAs in the gut are affected by dietary patterns; the consumption of processed meat, fried potatoes, fish, margarine, and coffee is positively associated with the levels of all fecal BAs, whereas muesli consumption has a negative association with the levels of all fecal BAs [6]. Furthermore, a 6-month randomized controlled-feeding study showed that a high-fat diet (HFD) caused an increase in the levels of total and free BAs compared to a low-fat diet (LFD) [7].

In the ileum, most conjugated BAs are reabsorbed and transported via portal blood to the liver, while the remaining BAs (<10%) are further metabolized by the gut microbiota to produce secondary BAs, including deoxycholic acid (DCA) and lithocholic acid (LCA) [8]. Metabolism from primary to secondary BAs consists of the following two steps: the hydrolysis of conjugated BAs and 7α/β-dehydroxylation of CA and CDCA to produce DCA and LCA [4,9]. Gut bile salt hydrolases (BSHs, EC 3.5.1.24) hydrolyze conjugated BAs to free primary BAs, which is the first step and gatekeeper of BA transformation in the gut [10]. The BSHs are affiliated with Firmicutes, Bacteroidetes, Actinobacteria, Proteobacteria, and Euryarchaeota [11,12]. *Lactobacillus* strains with BSH activity are a criterion for selecting probiotics with cholesterol-lowering and anti-obesity effects [13]. *Blautia obeum* can shape the chemical environment of the gut through its BSH activity, which can degrade taurocholate and reduce colonization of the major human diarrheal pathogen *Vibrio cholerae* [14]. Manipulation of the gut bacteria with BSH is considered a promising strategy to benefit human health [10,15].

Studies regarding BSHs related to host health are primarily performed using mouse models. The effect of BSHs on human health is currently unclear. Recently, we demonstrated that gut BSH abundance is significantly related to human diseases, including obesity and T2D as well as cardiovascular, liver, gastrointestinal, and neurological diseases [16]. Among these diseases, the risk of obesity, T2D, and cardiovascular diseases is closely linked to dietary patterns [17]. Increasing evidence has shown that plant-based diets are effective in preventing various chronic diseases [18]. However, dietary patterns of high calorie intake with high amounts of sugar and fat seem to increase the risk [19]. A ketogenetic diet (KD) is considered an effective treatment for obesity, diabetes, steatohepatitis, neurodegenerative disease, and cancer [20]. BA metabolism by gut microbes is also related to these conditions [21]. Considering the importance of BSHs in terms of health and the contribution of dietary patterns to human health, we performed a metagenome-wide association study to analyze diet-associated changes in BSH levels in the human gut microbiome. First, we collected publicly available datasets from three metagenomic studies with different dietary interventions and controls. Second, we evaluated the abundance of BSHs by mapping the BSH gene sequences to the gut metagenomic data. Finally, we investigated how the diversity and taxonomic changes in BSHs were affected by diet.

## 2. Results

### 2.1. Characteristics of the Metagenomic Datasets Used

The majority of studies regarding the effects of diet on the human gut microbiota have focused on the microbial community structure based on 16S rDNA analysis [22,23]. Compared to microbiota studies, metagenomics can produce data with both higher taxonomic resolution and gene functions with improved statistical precision [24]. However, as of May 2020, only three metagenomic sequencing studies have linked diet and human health (Table 1) [25,26,27]. The three studies performed controlled dietary interventions to compare the metagenomic difference between a baseline diet (BD) and a KD, an underfeeding diet (UFD) and an overfeeding diet (OFD), and a BD and a low-carbohydrate (CHO) diet (LCD). In the cohort treated with a KD, the inpatient crossover study was performed with 17 overweight or class I obese nondiabetic adult men who served as the BD (50% CHO, 15% protein, and 35% fat) for four weeks followed by a four-week KD (5% CHO, 15% protein, and 80% fat) [25]. Data for the UFD and OFD were collected from a randomized crossover inpatient dietary intervention in which all participants were treated with 3 days of over and underfeeding with a 3 day washout period in a random order [26]. Across both groups, the ratio of CHO:protein:fat in the diets was the same (50% CHO, 20% protein, and 30% fat), but the caloric intake of the OFD group was three times that of the UFD group. LCD analysis was conducted on a dataset of 10 subjects with obesity and high liver fat. The subjects were first fed a BD (40% CHO, 18% protein, and 42% fat) then served a LCD (4% CHO, 24% protein, and 72% fat) for 14 days [27]. The three datasets included information regarding both the different dietary intake amounts and the different components in each diet.

### 2.2. Abundance of BSHs in the Human Gut with Different Diets

**Figure 1:**
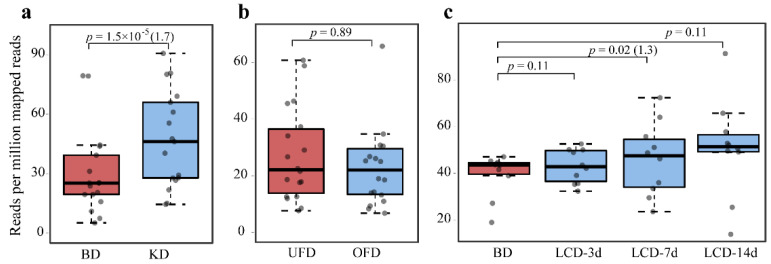
Changes in the total abundance of bile salt hydrolases in the human gut microbiome in (**a**) the baseline diet (BD) versus ketogenetic diet (KD) cohort, (**b**) underfeeding diet (UFD) versus overfeeding diet (OFD) cohort, and (**c**) BD versus low-carbohydrate diet (LCD) cohort. Statistical significance was calculated using the paired Wilcoxon test. The fold-change is shown in brackets after the *p* value.

We used 44 previously characterized BSHs (Supplementary Table S1) to search the UniProt protein database and selected the sequences from the human gut microbiota. Finally, 626 sequences were obtained from cultivated microorganisms and metagenome-assembled genomes from the human gut microbiota (Supplementary Dataset S1). We mapped the reads of the three datasets with different diets to the BSH sequences from gut microbes to analyze the differences in BSH abundance, which were indicated by both the *p* value and fold-change (FC) at *p* < 0.05 (Figure 1). BSH abundance under KD conditions increased significantly compared to that under BD conditions in the human gut (*p* = 1.5 × 10^−5^, FC = 1.7) (Figure 1a). The abundance of BSH in the human gut from subjects fed the OFD did not change significantly compared to that in subjects fed the UFD (*p* = 0.89) (Figure 1b), despite OFD treatment resulting in a significant decrease in total bacterial colonization levels [26]. We further compared BSH abundance in the BD and LCD cohorts (Figure 1c), and the results indicated that the abundance of BSHs also increased significantly during the 7 day study period (*p* = 0.02). In the 3 and 7 day study periods, the total abundance of BSHs did not show noticeable changes under LCD treatment (*p* = 0.11). Therefore, among the three dietary treatments tested in the study, only the KD significantly affected the abundance of BSHs.

### 2.3. Clustering of Gut BSHs and Abundance of Proteins from Each Cluster in Different Diets

**Figure 2:**
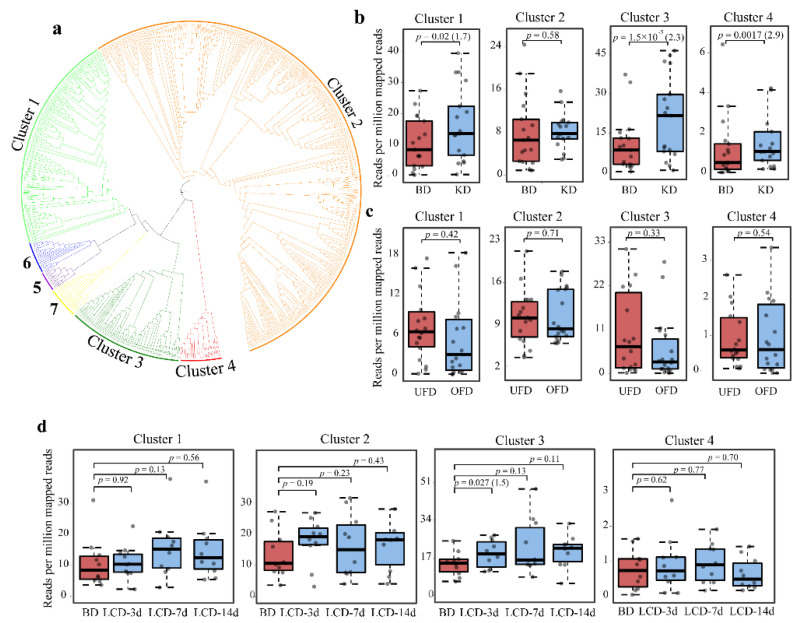
Classification of bile salt hydrolases (BSHs) and their abundance in each cluster in the human gut. (**a**) Classification and evolutionary relationships of BSHs. Maximum-likelihood phylogenetic tree for the BSHs listed in the Supplementary dataset S1 were generated using MEGA X. BSHs from different clusters were painted by the presented color. The abundance of the BSHs of Clusters 1 to 4 from the datasets of the baseline diet (BD) versus ketogenetic diet (KD) cohort (**b**), underfeeding diet (UFD) versus overfeeding diet (OFD) cohort (**c**), and BD versus low-carbohydrate diet (LCD) cohort (**d**) were further compared. The paired Wilcoxon test was used for statistical analysis. The fold-change is shown in the brackets after the *p* value if *p* < 0.05.

The protein sequence similarity network (SSN) of the gut BSHs was further generated with a criterion of >40% sequence identity, which separated the gut BSHs into seven clusters (Figure 2a and Supplementary Figure S1). The classification was in accordance with that of our previous study, and we named the clusters similarly to facilitate understanding [16]. The proteins from Clusters 1 and 3 were predominantly from Bacteroidetes, consisting of 136 and 71 proteins, respectively. There were 344 proteins in Cluster 2, which were mainly from Firmicutes and Actinobacteria. Proteins in other clusters consisted of <40 proteins from Firmicutes (Supplementary Figure S1). Sequence analysis demonstrated that only the proteins from Clusters 1 and 3 harbored N-terminal signal peptides (Supplementary Figure S2). Phylogenetic analysis showed that the proteins from Clusters 1 and 3 were localized in one clade in the tree with the proteins from Clusters 5, 6, and 7, which did not have the signal peptide. Otherwise, the proteins in Cluster 2 without signal peptides formed another separate clade. Cluster 4 was located away from the two clades in the phylogenetic tree (Figure 2a). These results suggest that the BSHs from one phylum may originate from different hydrolase precursors despite having common features in N-terminal sorting sequences.

Our previous study suggested that the abundance of BSHs from different clusters showed distinct relationships with human diseases [16]. In this study, we mapped the gut BSH sequences in each cluster to the datasets of different diets to evaluate the relationship. The abundance of BSHs from Clusters 1 to 4 was relatively high, whereas proteins from other clusters could not be detected (Supplementary Figure S3). We compared the abundance of BSHs from Clusters 1 to 4 between different dietary interventions and controls (Figure 2). For the KD versus BD, the BSH levels from Clusters 1, 3, and 4 increased significantly (*p* = 0.02, 1.5 × 10^−5^, and 0.0017, respectively; FC = 1.7, 2.3, and 2.9, respectively). In the OFD cohort, the BSH levels from the four clusters did not show a substantial difference. Finally, only the protein levels in Cluster 3 increased significantly at 3 days with the LCD (*p* = 0.027). There was no obvious trend of alteration in the abundance of BSH in other clusters in the LCD cohort. Overall, these results indicated that the relationship between the abundance of microbial BSHs in the gut and diet varies depending on different dietary patterns.

### 2.4. Taxonomic Diversity of BSHs in the Human Gut with Different Diets

**Figure 3:**
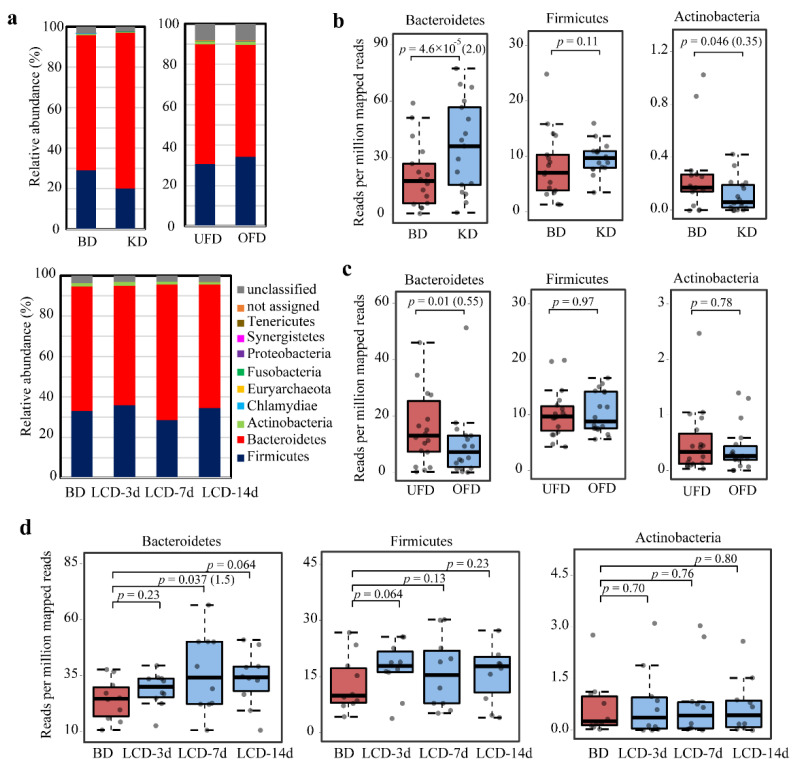
Taxonomic classification at the phylum level and relative abundance of bacteria with bile salt hydrolases (BSHs) in the human gut microbiome. (**a**) Taxonomic distribution of BSH-active bacteria at the phylum level in subjects of the baseline diet (BD) and ketogenetic diet (KD), underfeeding diet (UFD) and overfeeding diet (OFD), and BD and low-carbohydrate diet (LCD) after 3, 7, and 14 days (LCD-3d, LCD-7d, and LCD-14d). The abundance of the bacteria producing BSHs from Bacteroidetes, Firmicutes, and Actinobacteria were further compared as the BD versus KD cohort (**b**), UFD versus OFD cohort (**c**), and BD versus LCD cohort (**d**). Statistical significance was calculated using the paired Wilcoxon test. The fold-change is shown in the brackets after the *p* value.

BSHs in the gut are from 12 phyla but mainly belong to the two dominant gut phyla Bacteroidetes and Firmicutes [11]. The BSHs from the bacteria of these two phyla are considerably different in sequence; the proteins from Bacteroidetes contain N-terminal signal peptides, while those from Firmicutes do not harbor these peptides [16]. This sequence difference motivated us to examine how the community structure of BSH microbes is affected by diet. The results showed that the BSHs were predominantly from bacteria belonging to Bacteroidetes and Firmicutes in the three cohorts examined (Figure 3a). In the phylum Bacteroidetes, BSHs were mainly found at the genus level of *Alistipes*, *Bacteroides*, and *Barnesiella*. In the phylum Firmicutes, BSHs showed a wide distribution at the genus level, including *Ruminococcus*, *Eubacterium*, *Lachnospira*, *Oscillibacter*, *Clostridium*, *Mediterraneibacte*, *Holdemanella*, *Roseburia*, and *Lactobacillus* (Supplementary Figure S4). The phylum with the third-highest abundance of BSHs in the gut was Actinobacteria. We compared the abundance of BSHs from Bacteroidetes, Firmicutes, and Actinobacteria in the gut between different dietary interventions and controls. The abundance of BSHs from Bacteroidetes increased significantly in the KD cohort (*p* = 4.6 × 10^−5^, FC = 2.0) (Figure 3b). The abundance of BSHs from Firmicutes under the KD treatment did not show a significantly varying trend (*p* = 0.11). The abundance of BSHs from Actinobacteria was significantly reduced under KD treatment (*p* = 0.046, FC = 0.35). These results suggest that consuming a KD altered the community structure of BSH-active microbes significantly. For the UFD versus OFD, Bacteroidetes BSH levels decreased significantly (*p* = 0.01, FC = 0.55). No significant difference in the abundance of BSHs from Firmicutes and Actinobacteria was observed (*p* > 0.05) (Figure 3c). Although the constituents of the KD and LCD were similar, the LCD did not change the community distribution of BSH-active bacteria in the human gut (Figure 3d). Only the abundance of BSHs from Bacteroidetes increased significantly at 7 days following the onset of an LCD (*p* = 0.037). After 14 days, no significant difference in the abundance of BSHs from Bacteroidetes, Firmicutes, and Actinobacteria was observed between LCD and BD subjects (*p* > 0.05). Taken together, these data suggest that the alteration of BSHs was limited to the KD cohort; the UFD and LCD did not change the community structure of BSH-active bacteria.

## 3. Discussion

Dietary patterns and interventions affect the overall composition of the gut microbiome and exert direct effects on mammalian health. The effects of diet, including LFDs, HFDs, high protein diets, LCDs, very-low-calorie diets, and KDs, on the health and gut microbiota composition of animal models have been widely studied [28]. Diet is the key determinant of community structure and function of the human gut microbiota among several host-endogenous and -exogenous factors [29]. In the guts of both obese human subjects and animal models with HFD-induced obesity, the level of Bacteroidetes is lower and the level of Firmicutes is higher than in the respective lean control subjects [30]. A KD decreases the Firmicutes and Actinobacteria bacteria levels with a corresponding increase in Bacteroidetes bacteria levels in the guts of adults through the action of ketone bodies produced by the host [25]. The same alteration occurs in children with refractory epilepsy following adherence to a KD [31]. Gut microbial shifts on KDs reduces levels of intestinal proinflammatory Th17 cells [25], which may contribute to the efficacy of KD in improving glycemic control and reductions in body fat [32]. In addition, 3-OxoLCA, a secondary BA and downstream product from BSH catalysis, could inhibit the differentiation of TH17 cells also [32]. The present study showed that BSHs in the KD cohort were significantly altered, including the total abundance, abundance in different clusters, and abundance at the phylum level. The total abundance of BSHs in the KD cohort increased significantly. As the KD diet contained a high ratio of fat and protein, the results are in accordance with those of a previous study showing that an animal-based diet increased the human gut BSH abundance compared with a plant-based diet based on metatranscriptomics analysis [33]. The increased BSHs may increase the concentration of CA, the substrate of BSHs, which could promote weight loss in mice fed a HFD through increased metabolic rate in brown fat tissue mediated by TGR5 signaling pathways [34]. Furthermore, the increased BSHs also contribute to a reduction of serum cholesterol by decreasing cholesterol absorption through coprecipitation unconjugated BAs with cholesterol in the intestinal lumen [35]. Since the KD has been considered a strategy for weight loss in recent years [36], we propose that the increase in microbial BSHs due to the KD may be a contributing factor that facilitates weight loss. Our study indicates that the abundance of BSHs in Clusters 1 and 3 increased in the KD cohort. As BSH-active *Lactobacillus plantarum*, marketed as a probiotic, has beneficial effects on host health [37], we propose that BSH-active bacteria from Cluster 1 and 3 (Supplementary Dataset S1) could be an alternative and suitable probiotic as the BSHs in the two clusters increased significantly with a KD.

The BSHs from Clusters 1 and 3 were predominantly from the phylum Bacteroidetes. The abundance study based on taxonomic aspects further displayed a significant increase in the BSHs from Bacteroidetes but not Firmicutes in the KD cohort. However, this study of BSHs mainly focused on the enzymes from Firmicutes, as 39 were from Firmicutes out of the 44 experimentally characterized BSHs (Supplementary Table S1). Furthermore, both the current study and previous studies have shown that the amount of BSHs from Bacteroidetes is relatively higher than the enzymes from Firmicutes in the human gut [11,16]. We suggest that BSH research should shift from BSHs in Firmicutes to those in Bacteroidetes. The characterized enzymes from Bacteroidetes Clusters 1 and 3 included *Bacteroides thetaiotaomicron* and *Bacteroides ovatus* [12,38]. During screening of the BSH activity of 20 Bacteroidetes strains, the majority preferred tauro- to glyco-conjugated BAs as substrates [38]. The in vitro enzyme activity assay also indicated that the enzymes in Cluster 1 (UniProt ID: A0A3A6KI09) and 3 (A0A3A6KGT7) prefer deconjugated tauro-BAs [11]. As only a few enzymes have been experimentally characterized in Clusters 1 and 3, it is not yet known whether other proteins have a similar preference for substrates. The well-studied enzymes in Cluster 2, including those from Lactobacillus and Bifidobacterium, have a wide range of substrate preferences, including glyco-glyco-/tauro-, and tauro-BAs [39]. Further studies regarding BSHs from Clusters 1 and 3 should be undertaken to understand the catalytic characteristics.

The present study indicated that only the KD altered the total abundance of BSHs, the abundance in different clusters, and community structure, while the OFD did not cause any changes. As the KD cohort had a low ratio of CHO and high ratio of fat—while the ratio of CHO, protein, and fat was the same for both the UFD and OFD—we propose that the dietary constituents, but not the food intake amounts, exerted a detrimental effect on the diversity of BSHs from Firmicutes and Bacteroidetes in the human gut. However, the abundance of BSHs in the LCD cohort did not show obvious changes despite the LCD and KD having a similar CHO, protein, and fat ratio. This may be because the LCD study recruited obese patients with NAFLD, where dysbiosis had occurred and confounded the quantification of BSHs [40].

We separated BSH homologs from the UniProt database into seven clusters in a previous study [16]. These sequences were not only from the human gut microbiome but also from the genome of bacteria from soil or other environmental sources. The proteins in Clusters 1 and 3 in the study were from both Proteobacteria and Bacteroidetes; however, the human gut microbiota is composed primarily of the phyla Bacteroidetes or Firmicutes [41]. Recent advances in sequencing and algorithms in the human gut microbiome have provided sufficient knowledge regarding genome sequences; for example, 204,938 reference genomes from the human gut microbiome were reported in 2020 [42]. Therefore, in this study, we only analyzed the BSH homologs available in human gut microorganisms. These sequences were from cultivated microorganisms or metagenome-assembled genomes in the human gut. Although the total protein sequences decreased compared with previous studies, the mapping outcome was much more convincing as the query (BSHs) and target sequences (datasets of the diets) were limited in the same resources.

The human diet can be influenced by many habitual, demographic, environmental, social, and individual factors [43]. Rigorous long-term studies comparing diet using methodologies that preclude bias and confounding factors are difficult to perform and are unlikely to be carried out for many reasons [44]. In the present study, we collected three cohorts from studies involving both human dietary intervention and gut microbial metagenomic analysis; however, the sample sizes in the datasets were relatively small. We acknowledge that this is a limitation of the current study. Fortunately, the included studies were carefully controlled through inpatient treatment [25,26] or through daily instruction by a dietician [27]. Another limitation is that validation using independent study populations should have been performed, similar to our previous study [16,24], but this was not performed because there were insufficient numbers of cohort datasets available related to diet. Considering the careful design and strict control of these studies, we suggest that the analysis based on these datasets is reliable and reproducible, and high-quality trials of dietary interventions are needed to further assess their effect on BA metabolism.

## 4. Materials and Methods

### 4.1. Collection and Analysis of BSHs from the Human Gut Microbiota

The experimentally characterized enzymes were obtained from our previous study (Supplementary Table S1). These sequences were designated as query sequences to perform a BLAST analysis against the UniProt database (Version: 2020_02) with a cut-off e-value of 10^−5^, and the proteins from human gut microbiota were retained (Supplementary Dataset S1). The SSN and phylogenetic trees of BSHs from the gut microbiota were generated based on a previously described method [16].

### 4.2. Abundance Analysis of BSH Genes in the Datasets Related to Diets

Whole-genome sequencing datasets of the human fecal metagenomes related to diet and generated on Illumina platforms were downloaded from the NCBI SRA database (Table 1). Only data from non-antibiotic/probiotic-treated hosts and using an Illumina sequencing platform were selected for analysis. Low-quality reads were trimmed and the nucleotide sequences of gut microbial BSHs were mapped to the remaining high-quality sequencing reads using the Burrows–Wheeler alignment tool (version 0.7.17-r1194-dirty) [45]. The aligned reads were filtered using the SAMtools algorithms (version 1.9) to only retain the reads that showed a mapping quality number above 60 [46]. BEDtools was used to count the number of reads (version 2.27.1-dirty, https://bedtools.readthedocs.io/, accessed on 20 October 2020). The Kaiju program (version 1.7.3) was used to identify the taxonomic information of each read in the phylum rank [47]. The read counts of BSHs were normalized to reads per million (RPM) and visualized by boxplots using the ggplot2 package (version 3.1.5) in R.

### 4.3. Statistical Analysis

All statistical analyses were performed using R. The Shapiro–Wilk test was used to assess the normality of the abundance of BSHs. The paired Wilcoxon signed-rank test was used to test the significance of differences in BSH abundance between test and control subjects, as the data were not normally distributed. The FC based on the RPM value was calculated by dividing the value from test diet-fed subjects by the value from the control subjects.

## 5. Conclusions

In conclusion, we collected three datasets from human gut metagenomic sequencing studies related to diet. The abundance and community structure of the BSHs in the three cohorts were analyzed. The results showed that the KD significantly influenced the abundance and community structure of BSH-active bacteria, while overfeeding had no effect on BSH abundance. To our knowledge, this study is the first to demonstrate the relationship between diet and BSHs in the human gut, which may allow the manipulation of BA metabolism via diet for the benefit of human health.

## Figures and Tables

**Table 1:** Fecal metagenomic studies of dietary interventions included in this study.

Dataset (Accession Number)	Dietary Pattern	Diet Components (CHO:protein:fat)	Energy Intake (kcal/d)	Sample No.	Intake Days	Age	Gender Female (%)/Male (%)	BMI	Country	Sequencing Method
Ang et al. (SRP189794)	Baseline diet (BD)	50:15:35	NS	17	14	35.1 ± 7.3	0/100.0	25–35	USA	Illumina HiSeq 2500 & NovaSeq 6000
	Ketogenic diet (KD)	5:15:80	NS	17	14					
Basolo et al. (SRP229815)	Underfeeding diet (UFD)	50:20:30	1494 ± 211	18	3	18–50	37.0/63.0	32.8 ± 8.0	USA	Illumina NovaSeq S2
	Overfeeding diet (OFD)	50:20:30	4446 ± 547	18	3					
Mardinoglu et al. (SRP126014)	Baseline diet (CD)	40:18:42	2234 ± 221	10	NS	53.7 ± 3.6	20.0/80.0	34.1 ± 1.2	Sweden	Illumina NextSeq 500
	Low-carbohydrate diet (LCD)	4:24:72	3115 ± 441	10	14					

## Data Availability

Data sharing not applicable.

## Supplementary Materials

The following are available online at https://www.mdpi.com/article/10.3390/ijms22073652/s1, Table S1. Experimentally characterized bile salt hydrolases from literature. Figure S1. Protein sequence similarity network (SSN) of bile salt hydrolases from the human gut. Figure S2. Protein sequence similarity network (SSN) of bile salt hydrolases (BSHs) annotated by signal peptides. Figure S3. The abundance of the BSH of Cluster 5–7 from the datasets. Dataset S1: Uniprot ID of protein se-quences used to generate sequence similarity network. References [11,12,38,48,49,50,51,52,53,54,55,56,57,58,59,60,61,62,63,64,65,66,67,68] are cited in the supplementary materials.
