# Supplementary material for: Diet-Related Alterations of Gut Bile Salt Hydrolases Determined Using a Metagenomic Analysis of the Human Microbiome
Source: Int J Mol Sci. 2021 Apr 1;22(7):3652. doi: 10.3390/ijms22073652 (PMC8038126; doi:10.3390/ijms22073652)
Supplement: Supplementary file 1 [file ijms-22-03652-s001.zip › ijms-1118338-supplementary/Supplementary data/supplenmetary data3.pdf]

Supplementary Table S1 Experimentally characterized bile salt hydrolases from literature.

**Entry**	**Organism**	**Length**	**Cluster**	**References**
A0A3A6KI09	*Bacteroides sp.* AF35-22	351	Cluster1(S)	[11]
Q8A600	*Bacteroides thetaiotaomicron*	352	Cluster1(S)	[38]
Q1WR93	*Lactobacillus salivarius*	316	Cluster2	[48]
C7AQX8	*Lactobacillus salivarius*	324	Cluster2	[48]
C7AQY2	*Lactobacillus salivarius*	324	Cluster2	[49]
C7AQX9	*Lactobacillus salivarius*	325	Cluster2	[49]
J7H3P9	*Lactobacillus salivarius*	324	Cluster2	[50]
A0A0R2G1E1	*Lactobacillus plantarum*	324	Cluster2	[51]
Q06115	*Lactobacillus plantarum*	324	Cluster2	[52]
M1R991	*Lactobacillus plantarum*	328	Cluster2	[53]
B9V405	*Lactobacillus gasseri*	325	Cluster2	[54]
A0A1Y0E209	*Lactobacillus gasseri*	326	Cluster2	[55]
Q5FKM3	*Lactobacillus acidophilus*	325	Cluster2	[56]
Q5FK51	*Lactobacillus acidophilus*	325	Cluster2	[56]
A5VJH0	*Lactobacillus reuteri*	325	Cluster2	[57]
A0A1B3PS02	*Lactobacillus johnsonii*	326	Cluster2	[58]
A5HKP3	*Lactobacillus acidophilus*	316	Cluster2	[58]
A0A2M9NK78	*Lactobacillus johnsonii*	325	Cluster2	[58]
Q9F660	*Lactobacillus johnsonii*	326	Cluster2	[59]
P97038	*Lactobacillus johnsonii*	316	Cluster2	[59]
Q9KK62	*Bifidobacterium longum*	317	Cluster2	[60]
A0A087BL81	*Bifidobacterium longum*	317	Cluster2	[61]
Q6R974	*Bifidobacterium bifidum*	316	Cluster2	[62]
G0YYC2	*Bifidobacterium animalis*	314	Cluster2	[63]
Q53CP8	*Bifidobacterium animalis*	314	Cluster2	[64]
A0A072MTF9	*Bifidobacterium pseudocatenulatum*	316	Cluster2	[61]
P54965	*Clostridium perfringens*	329	Cluster2	[65]
C7CXJ5	*Enterococcus faecalis* T2	324	Cluster2	[66]
R6GBZ3	*Eubacterium hallii* CAG:12	324	Cluster2	[11]
A0A380KNN4	*Streptococcus infantarius*	325	Cluster2	[11]
D6DDI0	*Bifidobacterium longum*	334	Cluster2	[11]
A5ZA20	*Eubacterium ventriosum* ATCC 27560	329	Cluster2	[12]
A5ZWR0	*Blautia obeum* ATCC 29174	325	Cluster2	[12]
A5ZM11	*Blautia obeum* ATCC 29174	326	Cluster2	[12]

A4E835	*Collinsella aerofaciens* ATCC 25986	308	Cluster2	[12]
A7A5K1	*Bifidobacterium adolescentis* L2-32	347	Cluster2	[12]
A0A3A6KGT7	*Bacteroides sp.* AF35-22	361	Cluster3(S)	[11]
A7LYY3	*Bacteroides ovatus*	359	Cluster3(S)	[12]
A7V5S3	*Bacteroides uniformis*	361	Cluster3(S)	[12]
A0A416F798	*Clostridium sp.* AF32-7AC	336	Cluster4	[11]
H6WSA2	*Lactobacillus fermentum*	325	Cluster5	[67]
M1R367	*Lactobacillus plantarum*	317	Cluster6	[53]
A0A1F0S498	*Lactobacillus sp.* HMSC056D05	338	Cluster7	[68]
E2YQS1	*Enterococcus faecalis* DAPTO 512	355	Cluster7	[11]

Supplementary Fig. S1

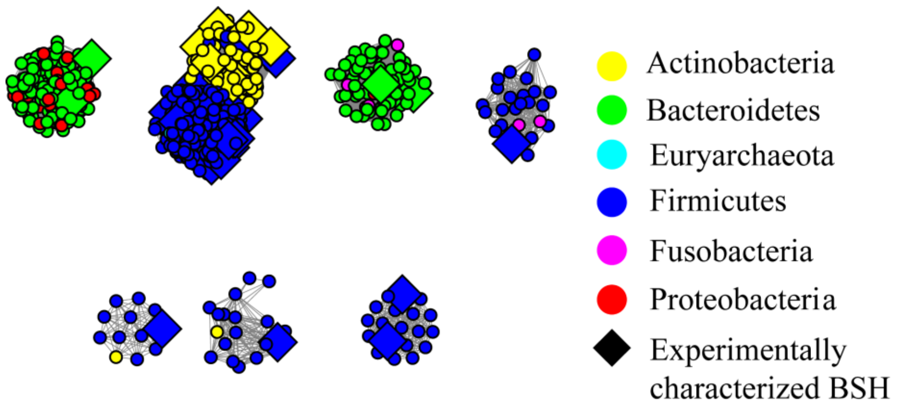

Supplementary Fig. S1 Protein sequence similarity network (SSN) of bile salt hydrolases from the human gut. The proteins listed in Supplementary dataset were used generate the network using an e-value threshold of 10^{-60} (>40% sequence identity). A representative node from each cluster listed in Supplementary Table S1 is enlarged. Nodes from the same phylum are represented by the same color as presented in the right part.

Supplementary Fig. S2

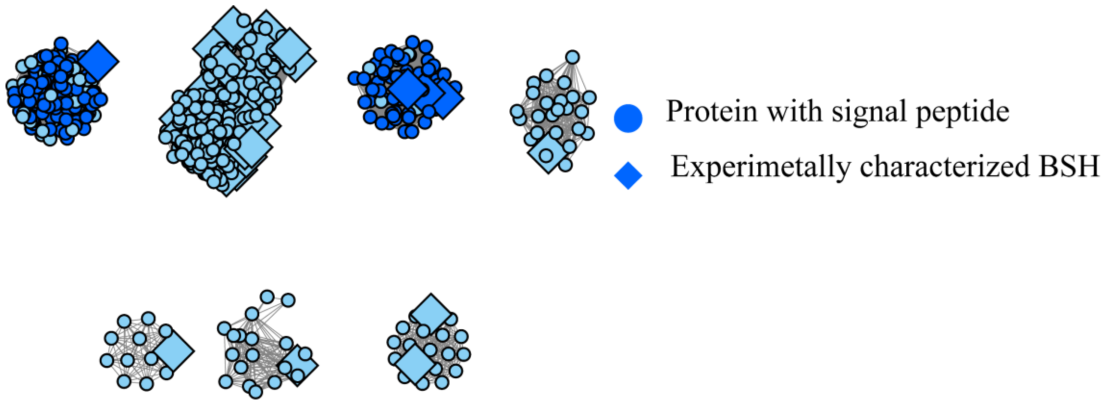

Supplementary Figure S2. Protein sequence similarity network (SSN) of bile salt hydrolases (BSHs) annotated by signal peptides. The proteins with signal peptides are shown in deep blue.

Supplementary Fig. S3

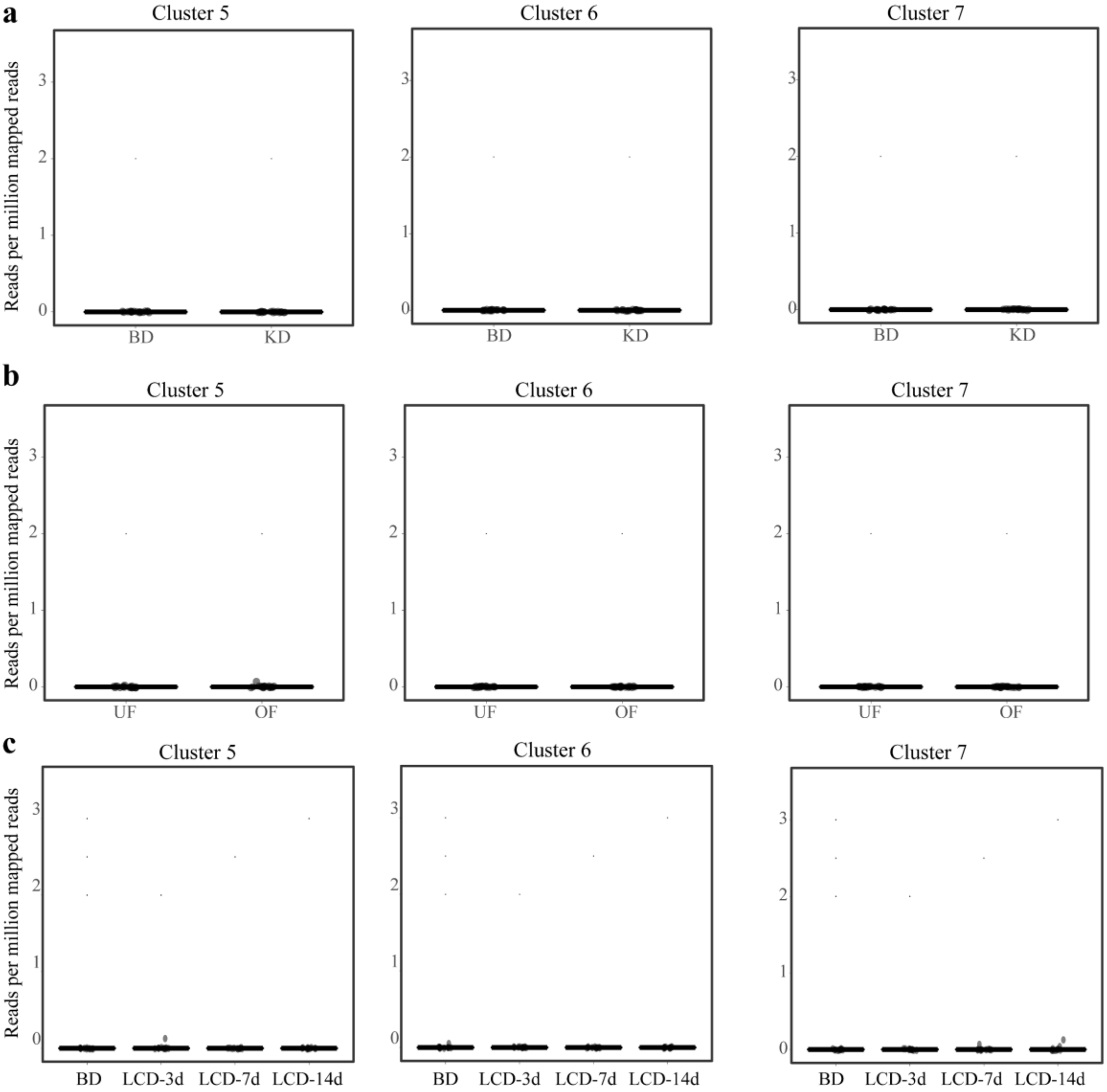

Supplementary Figure S3. The abundance of the BSH of Cluster 5-7 from the datasets of the BD versus KD cohort (b), UFD versus OFD cohort (c), and the BD versus LCD cohort (d) were further compared.

Supplementary Fig. S4

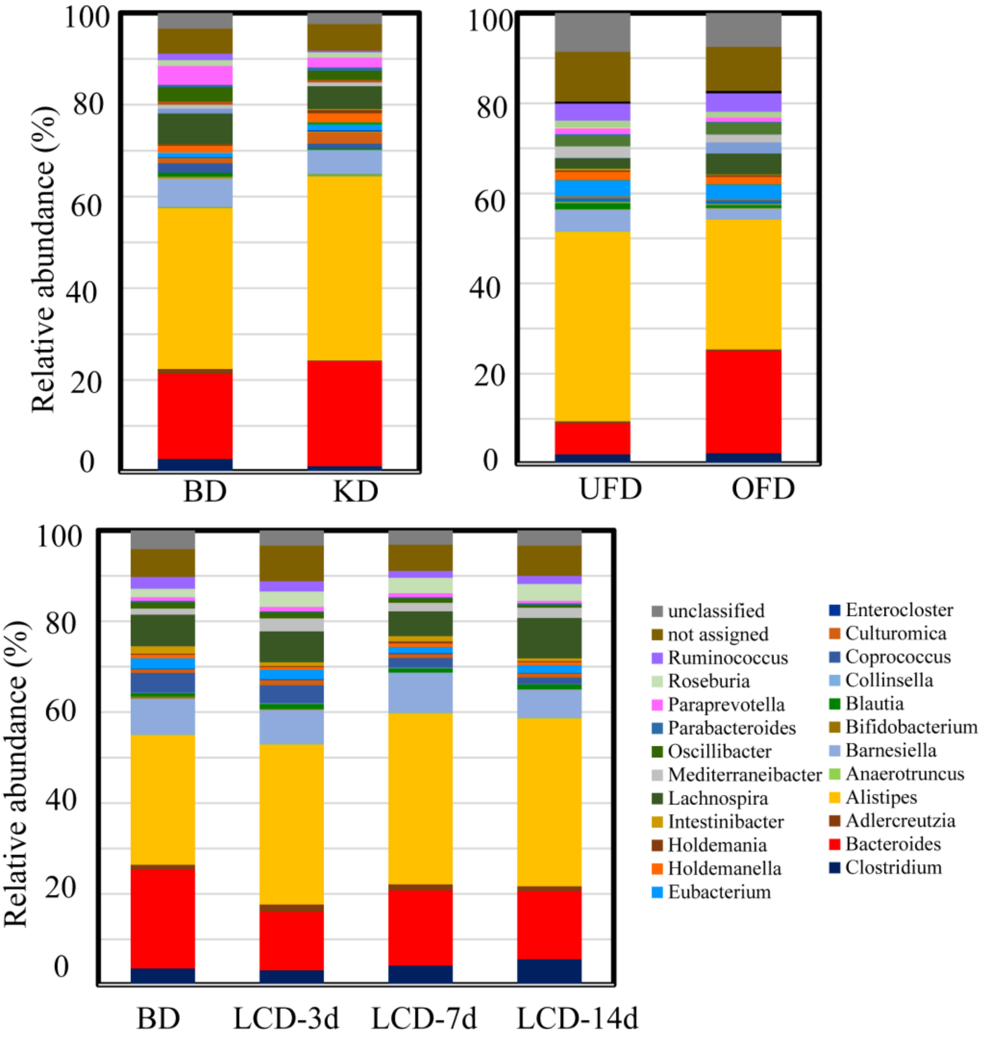

Supplementary Fig. S4 Taxonomic distribution of BSH-active bacteria at the genus level in subjects of the baseline diet (BD) and ketogenetic diet (KD), underfeeding diet (UFD) and overfeeding diet (OFD), and BD and low-carbohydrate diet (LCD) after 3, 7, and 14 days (LCD-3d, LCD-7d, and LCD-14d).
